# Can Cancellara Really be a *Flandrien*? Ethno-Cultural Identity Representation Predicts Regional Exclusivity of a Historically Contested Cycling Term

**DOI:** 10.5334/pb.358

**Published:** 2018-03-19

**Authors:** Stef Van Puyenbroeck, Pascal Delheye, Stijn Knuts, Liesbeth Vander Elst, Katrien Fransen, Norbert Vanbeselaere, Filip Boen

**Affiliations:** 1 Department of Movement Sciences, KU Leuven, Leuven, BE; 2 Department of Political Sciences, UGent, Gent, BE; 3 Faculty of Psychology and Educational Sciences, KU Leuven, Leuven, BE

**Keywords:** Cyclism, flandrien, ethno-cultural identity representation, Flemish identity, regional exclusivity

## Abstract

In Flanders the term *flandrien* refers to cyclists who display a strong work ethic, great perseverance, are powerful and who perform best in adverse weather conditions. Until the 1960s, only leading cyclists originating from the province of West- and East-Flanders were considered as *flandriens*. After 1960, the media extended the use of this term to Belgian cyclists in general and even to international cyclists. The present study examined whether Flemish citizens agree with this generalization considering that the term *flandrien* still plays a highly symbolical role in the public discourse on Flemish identity. First, the main aim was to investigate whether having an ethno-cultural identity representation of the Flemish identity is positively related to perceived regional exclusivity of the term *flandrien*. Second, this study explored whether Flemish identification moderates this relation (i.e. this relation is only expected for high identifiers) and also predicts Flemings’ regional exclusivity of the term. Results revealed that the more Flemings endorse an ethno-cultural identity representation the more they consider a *flandrien* as an exclusively Flemish cyclist, and the less they will include international cyclists in their consideration of a *flandrien*. Flemish identification did not moderate this relation but did predict the consideration of a *flandrien* as a Flemish cyclist. These findings indicate that the current interpretation of the historical cycling term *flandrien* is influenced by cultural conceptualizations of Flemish identity.

## Introduction

In the 2010 edition of the Tour of Flanders, the Swiss cyclist Fabian Cancellara accelerated with impressive power on the steep climb of the so-called ‘Wall of Geraardsbergen’, leaving everyone behind and winning Belgium’s most famous one-day cycling race. Just one week later, he sped over the cobbles of Paris-Roubaix to his second one-day classic victory in seven days. Mainly because of this exceptional double victory, Cancellara was honored with the ‘International Flandrien Award of 2010’ during the annual ‘gala of the *flandrien*’, a prestigious and popular show broadcasted live on Flemish television.

Historically, the term *flandrien* was reserved in the media for Flemish cyclists that were physically powerful, had a visibly high sportive work ethic and exhibited great perseverance while racing (Knuts et al., 2011). The generalization by the media of this originally purely Flemish term to an international cyclist raises two interesting questions. First of all, do Flemish citizens agree with this generalization of this strong symbol of Flemish identity? Second, does the way in which Flemings experience their Flemish identity influence their attribution of the term *flandrien* in terms of regional exclusivity? By trying to answer these questions, we aim to contribute to the understanding of the role that sports play in the formation and continuation of self-perceived regional identities.

### History of the cycling-term flandrien and its importance to Flemish identity

Worldwide, sports serve the function of creating and enhancing local, regional or national group identities. According to Maguire (1994), such collective identities are shaped and reshaped through a mixture of shared opinions, habits, beliefs and feelings. Specific sports, sports teams and sports performances are often constructed as expressions or symbols of local, regional or national identities (Vanreusel, 2006). For example, in Ireland the sport of rugby union serves as a powerful symbolic marker of Irish national identity. For the Irish, the physicality of the game is compatible with their temperament and is therefore seen as an important symbol of their identity as Irishmen (Tuck, 2003).

In Belgium, cycling has historically played a crucial role in the construction of group identities at different levels (i.e. local, regional and national) (Knuts & Delheye, 2011). This small Western-European country is historically marked by a complex interplay between different national identities. While its population mainly consists of two ethno-linguistic groups, a Dutch-speaking Flemish group and a French-speaking Walloon group, this diversity was not reflected by the country’s public life at its independence in 1830, its government and civil administration being unilingual French. Already from the 1840s onwards, a ‘Flemish movement’ reacted against this discriminating state of affairs. In this gradually growing movement, the idea of a distinct Flemish nation and identity gradually took shape, which in some cases – especially after World War One – led to political support among its more radical supporters for the independence of Flanders from Belgium. After a federalization of the national state to the benefit of its Flemish and French-speaking regions in recent decades, a broad majority of Flemings express a moderate, largely cultural – and rarely anti-Belgian – Flemish identity. At the same time, there is still a significant minority of separatist Flemish nationalists, united in political parties such as the right-wing *Nieuw-Vlaamse Alliantie* (N-VA), which has become the biggest Flemish party during the elections in 2014.

Being the first modern sport that was already adopted by the lower classes around 1900 (Delheye, Knuts, & Vanysacker, 2011), cycling became important in creating and enhancing this growing Flemish group identity from the Interwar period onwards (Knuts & Delheye, 2011). The introduction of the term *flandrien*, which started in West- and East Flanders (i.e. the Western part of Dutch-speaking Flanders), served to convey Flemish identity and pride during the interbellum, because the Dutch-speaking Flemings strived to become more autonomous from the French-speaking Walloons.

It should be noted that at the beginning of the twentieth century, the term *flandrien* had a rather negative connotation as shorthand description for the rude West- and East-Flemish seasonal workers in Wallonia and the Northern part of France (Derez, 2008; Musschoot, 2008). In the cycling world, this term was used for the first time by Karel Van Wijnendaele, a West-Flemish sports journalist and key figure of the influential sports newspaper *Sportwereld*. In 1913, he gathered a team consisting mainly of West- and East-Flemish track cyclists around him and began managing them in races in Belgium and abroad. With their aggressive, rough riding style, these cyclists first had themselves noticed during races on Brussels’ indoor cycling track (or *vélodrome*). The French-speaking press described these *flandriens* as savages, displaying unfair and aggressive behaviour. From this moment, the term *flandrien* became synonymous with the straightforward style of Flemish cyclists. The latter characteristics were further confirmed by finishing first, second and third at the ‘Six hours of Paris’ in 1913, again using their aggressive, perseverant style. Nevertheless, Van Wijnendaele consistently casted *flandriens* in a positive and even heroic light. He described ‘his’ *flandriens* as personifications of a supposed ‘essence’ of the Flemish people: poor, but strong and showing a lot of willpower. They lacked tactics, but won through strength and perseverance (Backelandt, Cornillie, & Vanwalleghem, 2006).

Importantly, the use of the term *flandrien* was initially restricted to East- and especially West-Flemish cyclists. In the 1900s, cycling was most popular in these two Belgian provinces. In West-Flemish ‘cycling-cities’ such as Roeselare, inhabitants were immensely proud of ‘their’ successful local cyclists. These cyclists served as idols and prototypes that created and enhanced a local identity amongst the inhabitants of these cities and, by extension, amongst the inhabitants of West-Flanders in general (Knuts & Delheye, 2011).

From the 1920s until the 1950s, the label *flandrien* was still exclusively used for West- and East-Flemish cyclists and their supposed great strength, perseverance and straightforward cycling style. However, their role as representatives was no longer restricted to these two Flemish provinces, as they became representatives of Flanders as a whole. The term *flandrien* also appeared in many other newspapers than Karel Van Wijnendaele’s *Sportwereld*. This *flandrien* title could especially be earned by winning the Tour of Flanders, a race founded by *Sportwereld* in 1913, and still one of the most famous one-day cycling classics today.

According to Van Wijnendaele, the aim of the Tour of Flanders was to make people proud of the Flemish region. East- and West-Flemish cyclists’ strong characteristics would be the perfect reflections of this region. The most typical *flandrien* of that time was the West-Flemish cyclist Albéric ‘Briek’ Schotte, who appeared to win his races solely on willpower. His permanent urge to attack, his victories in the Tour of Flanders, his suffering appearance, his skewed bending over his bike and the fact that he performed best in adverse weather conditions earned him the title of ‘the most prototypical *flandrien*’ (Backelandt, Cornillie, & Vanwalleghem, 2006).

After 1960, even though Schotte was still considered as the prototype of the *flandrien*, the provincial origin (i.e. being West- or East-Flemish) of a cyclist became less important to be considered as a *flandrien*. The use of the term expanded and in the 1980s and 1990s also non-West- or East Flemish (but still Flemish) cyclists, such as the ever-attacking Ludo Dierckxsens, were labelled as *flandriens*.

As the new millennium approached, even the Flemish origin became less important in order to be considered as a *flandrien* by the Flemish media. This was illustrated by the recent documentary *The Flandriens* (Canvas, 2010), in which the legendary Eddy Merckx, who grew up in Brussels, a non-Flemish region of Belgium were the majority speaks French, was presented as a *flandrien*. An even more poignant illustration of this trend was the fact that Philippe Gilbert won the trophy ‘*flandrien* of the year 2011’, despite his Walloon origin. More recently, even non-Belgian cyclists are labelled and honoured as *flandriens*. This is further illustrated by the fact that in 2003, 2006 and 2007, the trophy ‘*flandrien* of the year’ was awarded to Italian cyclist Paolo Bettini.

Considering this historical shift in how the media have dealt with the regional exclusivity of the term *flandrien*, the present study will question if and to what extent Flemish *citizens* agree with this labelling of foreign cyclists as *flandriens*. Nowadays, *flandriens* are still considered as important symbols of a Flemish identity (Knuts et al., 2011; Knuts & Delheye, 2014). The attributes and characteristics of a *flandrien* have always been presented as an important part of the prototype of Flemish people. This becomes especially clear during the Tour of Flanders. Although this is an international race, it is considered as ‘Flemish cultural heritage’ of which Flemings should be proud. Its widespread popularity in Flanders is illustrated by the fact that on April 6^th^ 2014, the broadcast of this race reached an impressive market share of 71% in Flanders. Moreover, several observable expressions and symbols emphasize that a clear sense of Flemish identification is activated during this race. One of them is the abundant usage of the term *flandrien*, the Flemish flag (i.e. black lions on a yellow background), and the descriptions of heroic performances of previous *flandriens* in the Flemish media. This creates a feeling of ‘us (i.e. the Flemings) versus them (i.e. all the rest)’ and symbolizes Flemish identity (Vanreusel, 2006).

### Different interpretations of a flandrien and the role of identity representations

According to the Social Identity Approach (SIA: Haslam, 2004), the self-concept consists of both a personal identity and a social identity, which is based on group membership. Each group is associated with an in-group prototype, which refers to the attributes, behaviours, norms and values that are characteristic for that group. The more strongly individuals identify with a group, the more they tend to live up to the prototype of this group and internalize its corresponding norms and values. For example, it has been shown that the more strongly hockey fans identify as fans of a certain team, the more they internalize the norms with respect to out-group derogation (Amiot, Sansfaçon, & Louis, 2014).

In terms of SIA, the term *flandrien*, often described as a powerful symbol of Flemishness, can be considered as a part of the in-group prototype of Flemish identity. In times that are characterized by an increasing popularity of Flemish, nationalist right-wing political parties, who emphasize the uniqueness of the Flemings and Flanders, we raise the question whether all Flemings agree with the fact that international cyclists are now also considered as *flandriens* by the sports media.

It should be noted that the conclusions of the previous section were based on historical and sociological studies, which used contemporary primary sources (newspapers, periodical publications) and secondary literature. By contrast, in a recent social-psychological study Knuts et al. (2011) interviewed Flemings to investigate their perception of a *flandrien*. These authors found that only 29% accepted that also international cyclists could be considered as *flandriens* while a large majority (71%) disagreed with this statement. Importantly, this means that a large group of Flemings was not inclined to consider international cyclists as *flandriens*, even if these cyclists exhibited the same characteristics as a prototypical *flandrien* like Briek Schotte. These findings suggest that there are interindividual differences in the interpretation of a *flandrien* in terms of regional exclusivity, and that a large proportion of Flemings do not share the most recent media depiction of a *flandrien*.

However, this study only included a limited sample size (*N* = 203) and did not test whether the proportion of Flemings who have a regionally exclusive view on a *flandrien* (71%) significantly differed from the proportion of Flemings who are less restrictive and do not limit their definition of a *flandrien* in terms of regional exclusivity (29%). Furthermore, this study did not include variables that allowed the authors to directly probe into the possible underlying reasons for Flemings’ interpretation of a *flandrien*.

Considering that a *flandrien* functions as a symbol and prototype of Flemish identity, we propose that socio-psychological factors can explain these differences and can therefore influence Flemings’ interpretation of the term *flandrien*. One of these factors is the representation of national identity in terms of ethnic or cultural characteristics (Kohn, 1944; Smith, 2001). An ethnic identity representation defines group membership in terms of ancestral origin and genealogical grounds. Such a representation implies that group boundaries are impermeable for those with a different heritage or origin. Research in social psychology has revealed that individuals with an ethnic identity representation consider static identity components, including cultural traditions and symbols, as core aspects of their identity that need protection against change. Moreover, various studies also revealed that an ethnic identity representation can be related to anti-immigrant attitudes and social discrimination (e.g., Meeus et al., 2010; Pehrson, Brown, & Zagefka, 2009; Pehrson, Vignoles, & Brown, 2009).

Similarly, a cultural identity representation defines group membership in terms of sharing a common culture. From this perspective, individuals can join a group after adopting the dominant culture of that group and, most importantly, are expected to protect that culture in the future against change (Kymlicka, 2001). This cultural identity representation considers cultural traditions and symbols as core aspects of their identity, which need to be protected against change (Kohn, 1944; Smith, 2001).

Both cultural and ethnic identity representations value the cultural aspect of identity, and stress the importance of protecting this identity against change. Therefore, an ethno-cultural identity representation, as a combination of ethnic and cultural identity representations, can be considered as a conservative identity representation that especially stresses the anti-change aspect of identity. Individuals who endorse an ethno-cultural identity representation will be resistant against changes in the composition of their group in terms of its ancestral origins and cultural aspects or symbols.

## The Present Study

A first aim of the present study was to replicate Knuts et al.’s (2011) observed distribution of Flemings’ interpretation of a *flandrien* in terms of regional exclusivity in a larger sample. Based on findings from Knuts et al. (2011) and the historical importance of the term for the Flemish identity, we hypothesized that the majority of the Flemish have a regionally exclusive view on a *flandrien*, namely that Flemings tend to disagree with the statement that a *flandrien* can be considered as an international cyclist (Hypothesis 1).

The second and main aim was to address the absence of an underlying explanation of Flemings’ interpretation of a *flandrien*. More specifically we wanted to investigate the relation between the national identity representation of Flemings and their interpretation of this historical cycling term as an important symbol of Flemish identity. Because a *flandrien* is an important, historically grounded cultural symbol for the Flemish identity, we hypothesized that the more strongly Flemings endorse an ethno-cultural identity representation of Flanders, the more they would be inclined to protect the meaning of a *flandrien* against change and would therefore show more regional exclusivity in their interpretation (Hypothesis 2). More specifically, the more Flemings endorse an ethno-cultural identity representation, the more they consider a *flandrien* as an exclusively Flemish cyclist (Hypothesis 2a) and the less they would include international cyclists in their consideration of a *flandrien* (Hypothesis 2b).

As a third aim, we wanted to examine the role of Flemish identification in the interpretation of a *flandrien*. First, it is known that ingroup identification positively predicts ingroup favouritism (e.g., Brewer, 1999; Castano et al., 2002). Accordingly, we predicted that the more Flemings identify themselves as Flemish, the more they restrict a *flandrien* to Flemish cyclists in order to protect this historically important and positively valued term (Hypothesis 3). It should be noted that previous research did not show a straightforward relation between ingroup identification and outgroup derogation (e.g., Pehrson, Vignoles & Brown, 2009; Wagner et al., 2012). We therefore did not expect a relation between Flemish identification and the explicit exclusion of international cyclists as *flandriens*.

Second, it can also be expected that Flemish identification moderates the relation between an ethno-cultural identity representation and the interpretation of a *flandrien* in terms of regional exclusivity (Hypothesis 4). As mentioned before, identification with a group is necessary in order to attach value to the norms and values of its prototype. Moreover, Pehrson, Vignoles, and Brown (2009) reported that the combination of high identification and an ethnic identity representation resulted in the highest levels of negative out-group attitudes. In other words, the higher the identification with a group, the stronger the influence of identity representations on evaluations of outgroup members. It can therefore be predicted that when individuals identify only weakly as a Fleming, the extent to which they adhere to an ethno-cultural identity representation will not influence their perceived regional exclusivity of a *flandrien*. As opposed to low Flemish identifiers, it can be hypothesized that the extent to which high Flemish identifiers adhere to an ethno-cultural identity representation is positively related to the perception of the regional exclusivity of a *flandrien*. More specifically, the more strongly high Flemish identifiers adhere to an ethno-cultural identity representation, the more they will consider a *flandrien* as an exclusively Flemish cyclist (Hypothesis 4a) and the less they will include international cyclists in their consideration of a *flandrien* (Hypothesis 4b).

## Method

### Procedure

Participants were recruited by means of a standardized message. This message stated that the KU Leuven was investigating how cycling fans interpret the term *flandrien*. A link referring to the web-based questionnaire was included. Participants were informed that completing the questionnaire would take 20 to 25 minutes and they were asked to give their honest, personal opinion.

This message was spread through different channels. First, this message was sent via e-mail to 285 cycling clubs. The selected cycling clubs were either located in a cycling-specific region (i.e. regions with a rich cycling culture: Roeselare, Oudenaarde, and Herentals) or in a cycling-nonspecific region (Ieper, Sint-Niklaas, and Mechelen). Second, staff members of cycling events, administrators of cycling-related and tourism websites (Tourism Flemish Ardennes, Tourism East-Flanders, Flemish cycling federation, etc.) were contacted. Third, the message was published in the free newspaper *De Zondag* (2012, March 4, p. 50), printed each Sunday on more than 600,000 copies. Fourth, social media (*Facebook, Google+* and *Twitter*) were used to distribute the message. Fifth, the message was printed on 500 flyers, which were distributed in various places: in Mechelen on March 24, 2012, in Oudenaarde on March 31, 2012 (during the Tour of Flanders for leisure cyclists) and in Schoten, Lille and Herentals on April 4, 2012 (during the cycling race ‘Scheldeprijs’).

### Participants

In total, 2010 participants completed the web-based questionnaire, which was online from February 14, 2012 until April 16, 2012. Participation was voluntarily and participants could end the questionnaire at every moment. Anonymity was ensured and an e-mail address of one of the researchers was provided in case participants had any questions or remarks.

### Measurements

Table 1 provides a detailed overview of all variables used in this study, including their descriptive statistics and psychometric properties.

**Table 1:** Psychometric properties of the study variables.

Variable	Range	*M* or %	*SD*	α

Gender				
Female		16.1%		
Male		83.9%		
Age	13.00–89.00	40.66	16.59	
Education				
Lower education		49.9%		
Higher education		50.1%		
Fandom	1.00–5.00	4.35	0.95	
Ethno-cultural identity representation	1.00–5.00	3.52	0.87	.73
Flemish identification	1.00–5.00	3.77	0.97	.91
Consideration of a *flandrien* as a Flemish cyclist	1.00–5.00	3.55	1.37	
Inclusion of international cyclists as *flandriens*: one-item scale	1.00–5.00	3.09	1.47	
Inclusion of international cyclists as *flandriens*: cyclists list	1.00–5.00	2.52	1.03	.83

*Demographic variables*. The questionnaire assessed respondents’ age (in years), gender and education. With respect to education, respondents had to indicate whether they had a degree in lower education, lower secondary education, higher secondary education, higher, non-university education or university education.

*Cycling fandom*. To assess respondents’ level of cycling fandom, they had to indicate the extent to which they agree with the statement that they consider themselves as cycling fan on a 5-point Likert scale, ranging from 1 (*not applicable at all*) to 5 (*totally applicable*).

*Flemish identification*. Identification with the Flemish in-group was assessed through three items (i.e. ‘Being Flemish is very important to me’, ‘I am very proud to be Flemish’, ‘I feel strongly connected with other Flemings’) on a 5-point Likert scale, ranging from 1 (*total disagreement*) to 5 (*total agreement*). This scale was based on a six-item affective in-group identification scale from Boen et al. (2008). The internal consistency of this scale proved to be excellent (α = .91).

*Ethno-cultural identity representation*. The questionnaire included eight items that assessed the Flemish identity representation. All items were assessed on a 5-point Likert scale, ranging from 1 (*total disagreement*) to 5 (*total agreement*). Two items that assessed the desire for more autonomy for Flanders at the expense of the Belgian state (i.e. ‘I am pro more Flemish autonomy at the expense of the Belgian state’, ‘I am pro more Flemish independence at the expense of the Belgian state’). The other six items were part of the citizenship representation scales from Reijerse et al. (2012), who based their scales on Meeus et al. (2010): Two items were part of their three-item ethnic citizenship representation scale (i.e. ‘One can only be Flemish if he/she has Flemish (grand) parents’, ‘A Fleming is somebody who is born in Flanders’), two items were part of their six-item cultural citizenship representation scale (i.e. ‘A Fleming is somebody who lives according to the Flemish culture (language, habits, norms and values, etc.)’, ‘A Fleming protects the Flemish culture against multicultural influences’) and finally, two items were part of their five-item civic citizenship representation scale (i.e. ‘Flemish citizenship is allowed for everybody who settles legally in Flanders, lives by the law and participates actively in society’, ‘A Fleming accepts that other cultural groups have a right to the same social, political and cultural facilities as her/himself’).

An Exploratory Factor Analysis was conducted to identify the underlying structure of these eight items. After performing Direct Oblimin rotation, both the method of Cattell (1966; i.e. based on the scree plot) and the Kaiser eigenvalue-greater-than one rule (Kaiser, 1960) suggested the extraction of three components. Together, these components explained 69% of the total variance. An item was retained to construct a component when it had a minimum loading of .40, without having a cross loading higher than .40 (Table 2).

**Table 2:** Factor loadings for Exploratory Factor Analysis with Oblimin Rotation of items, based on the citizenship scales from Reijerse et al. (2012) and the autonomy items.

	Components		
	1	2	3

One can only be Flemish if he/she has Flemish (grand) parents.	**.79**	–.04	–.14
A Fleming is somebody who is born in Flanders.	**.83**	–.16	.02
A Fleming is somebody who lives according to the Flemish culture. (Language, habits, norms and values, etc.).	**.67**	.14	.23
A Fleming protects the Flemish culture against multicultural influences.	**.56**	.34	–.17
Flemish citizenship is allowed for everybody who settles legally in Flanders, lives by the law and participate actively in society.	.11	.15	**.82**
A Fleming accepts that other cultural groups have a right to the same social, political and cultural facilities as him/herself.	–.14	–.19	**.74**
I am pro more Flemish autonomy at the expense of the Belgian state.	–.04	**.95**	.05
I am pro more Flemish independence at the expense of the Belgian state.	–.01	**.94**	–.04

Factor loadings > .40 are in boldface.

The first component consisted of the four items that were part of the ethnic and cultural citizenship representation scale from Reijerse et al. (2012). The second component consisted of the two items that assessed the desire for more autonomy for Flanders at the expense of the Belgian state. The third component consisted of the two items that were part of the civic citizenship identification scale of Reijerse et al. (2012). The first component shows that there is an overlap between an ethnic and cultural identity representation. For this reason, the four items that loaded highly on the first component were combined and used as measurement of an ethno-cultural identity representation (component 1 in Table 2). The internal consistency of this ethno-cultural scale was acceptable (α = .73). The other four items were not used for further analyses in this study.

*Regional exclusivity of the term flandrien: consideration of a flandrien as a Flemish cyclist*. The item ‘A *flandrien* is a Flemish cyclist’ assessed the degree to which a *flandrien* is considered to be a Flemish cyclist. This item was assessed on a 5-point likert scale, ranging from 1 (*total disagreement*) to 5 (*total agreement*).

*Regional exclusivity of the term flandrien: inclusion of international cyclists as flandriens*. Two measurements were constructed to assess whether respondents also included international cyclists in their consideration of a *flandrien*. First, respondents had to rate the item ‘A *flandrien* can also be a foreign cyclist’ on a 5-point likert scale, ranging from 1 (*total disagreement*) to 5 (*total agreement*). Second, the questionnaire also contained eight international cyclists. Respondents had to indicate the extent to which they considered these cyclists as a *flandrien* on a 5-point likert scale ranging from 1 (*no flandrien at all*) to 5 (*totally a flandrien*). Four international cyclists were selected (i.e. ‘Fabian Cancellara’, ‘Bernard Hinault’, ‘Paolo Bettini’, ‘Thor Hushovd’) because they were expected to be best known amongst Flemings and contain prototypical *flandrien*-features such as a straightforward, fierce cycling style, winning on willpower and demonstrating a lot of perseverance. Therefore, for each participant, the average score for these four international cyclists was calculated. This combined score served as another, less abstract measurement of the inclusion of international cyclists as *flandriens*. This measurement demonstrated a good internal consistency (α = .83).

## Results

First, in order to test Hypothesis 1 and to compare our results with those from Knuts et al. (2011), it was necessary to trichotomize the scores on the item ‘A *flandrien* can also be a foreign cyclist’. Respondents who gave a rating of 1 or 2, were assigned to the category ‘disagree’, while respondents who rated this item 4 or 5 were assigned to the category ‘agree’. Participants who gave a rating of 3 were considered as ‘non-deciders’. This procedure resulted into three categories that all contained a proportion of the study sample: a ‘disagree’ category (*N* = 731), an ‘agree’ category (*N* = 972) and a ‘non-decider’ category (*N* = 298). To test Hypothesis 1, we compared the number of respondents who agreed with the statement that a *flandrien* can also be a foreign cyclist with the number of respondents who disagreed with this statement. In line with Hypothesis 1, a Chi-Square statistic indicated that the non-agreement category contained significantly more respondents than the agreement category (*χ*^2^ (1, *N* = 2204) = 34.11, *p* < .001). That is, there are more Flemings who adhere a regionally exclusive definition of a *flandrien* than Flemings who opine that a *flandrien* can also be an international cyclist.

In order to test whether this distribution fully replicates the findings from Knuts et al. (2011), an additional Chi-Square test was conducted. Combining data from the present study and the study from Knuts et al. (2011), we constructed a 2 × 2 contingency table in which the columns represented the sample (i.e. the present study sample vs. the study sample from Knuts et al. (2011)) and the rows the agreement vs. disagreement category (with the statement that a *flandrien* can also be an international cyclist). Results demonstrated that the proportion of disagreeing respondents was significantly lower in the present study as compared to the study from Knuts et al. (2011) (*χ*^2^ (1, *N* = 1906) = 14.36, *p* < .001). To summarize, although these results confirm Hypothesis 1, we did not fully replicate the exact distributions from Knuts et al. (2011) of Flemings who agree vs. disagree with the fact that a *flandrien* can also be an international cyclist.

Second, multiple hierarchical linear regression analyses were conducted to test Hypotheses 2–4. Table 3 provides an overview of the predictor variables, the dependent variables and their inter-correlations.

**Table 3:** Summary of intercorrelations for the study variables.

		1	2		3		4		5		6		7		8		9	

**Independent variables**																		
1.	Ethno-cultural identity representation	–	.49	**	.24	**	–.15	**	–.02		.14	**	.03		–.20	**	.03	
2.	Flemish identification		–		.19	**	–.10	**	.02		.17	**	.04		–.15	**	.03	
**Dependent variables**																		
3.	Consideration of a *flandrien* as a Flemish cyclist				–		–.65	**	–.43	**	.13	**	–.22	**	.01		–.10	**
4.	Inclusion of international cyclists as *flandriens*: one-item scale						–		.61	**	–.12	**	.26	**	–.03		.14	**
5.	Inclusion of international cyclists as *flandriens*: cyclists list								–		.05	*	.36	**	–.10	**	.18	**
**Control variables**																		
6.	Age										–		–.14	**	–.01		.02	
7.	Fandom												–		–.10	**	.29	**
8.	Education (0 = low, 1 = high)														–		–.08	**
9.	Gender (F = 0, M = 1)																–	

*p < .05, **p < .001.

The multiple hierarchical regression analysis was conducted for both operationalizations of regional exclusivity of the term *flandrien* (i.e. consideration of a *flandrien* as a Flemish cyclist and inclusion of international cyclists as *flandriens*). As opposed to the previous analysis of Hypothesis 1, all following analyses used the full range of likert-scale scores.

The procedure of the multiple hierarchical regression analysis was as follows. In step 1, the background variables gender, age, cycling fandom and education were entered. The variable education was dichotomized in a higher (1) versus no higher education group (0). In step 2, ethno-cultural identity representation and Flemish identification were included to test their contribution to the consideration of a *flandrien* as a Flemish cyclist and the inclusion of international cyclists in the consideration of a *flandrien*. In step 3, the interaction term between ethno-cultural identity representation and Flemish identification was entered. Ethno-cultural identity representation and Flemish identification were mean centered, following the recommended procedures for testing interactions in multiple regressions (Aiken & West, 1991; Jaccard, Turrisi, & Wan, 1990). The results of all regression analyses can be found in Tables 4, 5, 6.

**Table 4:** Hierarchical regression results predicting consideration of a flandrien as a Flemish cyclist.

Predictor	Consideration of a *flandrien* as a Flemish cyclist					
	Step 1 β		Step 2 β		Step 3 β	

Age	0.10	***	0.06	**	0.06	**
Gender (0 = F; 1 = M)	–0.05		–0.05	*	–0.05	*
Education (0 = low; 1 = high)	0.00		0.06	*	0.06	*
Fandom	–0.19	***	–0.20	***	–0.20	***
Ethno-cultural identity representation			0.21	***	0.21	***
Flemish identification			0.08	**	0.08	**
Ethno-cultural × Flemish identification					–0.00	
*R*^2^	.06		.12		.12	

*p < .05, **p < .01, ***p < .001.

**Table 5:** Hierarchical regression results predicting inclusion of international cyclists as flandriens as measured by the one-item scale.

Predictor	Inclusion of international cyclists as *flandriens*: one–item scale					
	Step 1 β		Step 2 β		Step 3 β	

Age	–0.08	***	–0.05	*	–0.05	*
Gender (0 = F; 1 = M)	0.06	**	0.07	**	0.07	**
Education (0 = low; 1 = high)	–0.02		–0.06	*	–0.06	*
Fandom	0.24	***	0.25	***	0.25	***
Ethno–cultural identity representation			–0.15	***	–0.15	***
Flemish identification			–0.03		–0.04	
Ethno–cultural × Flemish identification					–0.02	
*R*^2^	.08		.11		.11	

*p < .05, **p < .01, ***p < .001.

**Table 6:** Hierarchical regression results predicting inclusion of international cyclists as flandriens as measured by the cyclists list.

Predictor	Inclusion of international cyclists as *flandriens*: cyclists list					
	Step 1 β		Step 2 β		Step 3 β	

Age	0.11	***	0.12	***	0.12	***
Gender (0 = F; 1 = M)	0.07	**	0.07	**	0.07	**
Education (0 = low; 1 = high)	–0.05	*	–0.06	**	–0.06	**
Fandom	0.35	***	0.35	***	0.35	***
Ethno–cultural identity representation			–0.06	*	–0.06	*
Flemish identification			0.01		0.01	
Ethno–cultural × Flemish identification					–0.01	
*R*^2^	.15		.15		.15	

*p < .05, **p < .01, ***p < .001.

### Consideration of a flandrien as a Flemish cyclist

The results of the first step showed that the background variables explained 6% of variance in the consideration of a *flandrien* as a Flemish cyclist (*R*^2^ = .060, *F*(4, 1780) = 28.61, *p* < .001). Step 2 demonstrated that, when the background variables were statistically controlled for, both ethno-cultural identity representation and Flemish identification contributed significantly to the consideration of a *flandrien* as a Flemish cyclist (*R*^2^ = .122, Δ*R*^2^ = .06, Δ*F*(2, 1778) = 62,56 *p* < .001). The results of step 3 revealed that the interaction term between ethno-cultural identity representation and Flemish identification did not contribute significantly to the consideration of a *flandrien* as a Flemish cyclist (*R*^2^ = .122, Δ*R*^2^ = .00, Δ*F*(1, 1777) = 0.03, *p* = .87), which contradicts Hypothesis 4a. Therefore, only the regression coefficients after step 2 were interpreted.

First, gender (β = –.05, *p* = .041), cycling fandom (β = –.20, *p* < .001), education (β = .06, *p* = .013) and age (β = .06, *p* = .006) were revealed as significant predictors. In particular, men consider a *flandrien* less as a Flemish cyclist than women, and older and higher educated individuals consider a *flandrien* more as a Flemish cyclist than younger and lower educated individuals respectively. Moreover, the more strongly respondents claim to be cycling fans, the less they consider a *flandrien* as a Flemish cyclist.

Second, in line with Hypothesis 2a, ethno-cultural identity representation was a significant positive predictor of the consideration of a *flandrien* as a Flemish cyclist (β = .21 *p* < .001). In other words, the more strongly Flemings endorse an ethno-cultural identity representation, the more they consider a *flandrien* as a Flemish cyclist. Third, the same relation was found regarding Flemish identification (β = .08 *p* = .002). That is, the more Flemings identify themselves as Flemish, the more regionally exclusive their interpretation of a *flandrien* in terms of considering a *flandrien* as a Flemish cyclist, which confirmed Hypothesis 3a.

### Inclusion of international cyclists as flandriens: one-item scale

In the first step the background variables accounted for 8.3% of variance in the item ‘A *flandrien* can also be a foreign cyclist’ (*R*^2^ = .083, *F*(4, 1775) = 40.17, *p* < .001). The results of step 2 revealed that, when statistically accounting for the background variables, including ethno-cultural identity representation and Flemish identification contributed significantly to the prediction of the inclusion of international cyclists in the consideration of a *flandrien* (*R*^2^ = .109, Δ*R*^2^ = .026, Δ*F*(2, 1773) = 26.06, *p* < .001). The results of step 3 showed that the interaction term did not contribute significantly to the inclusion of international cyclists as *flandriens* (*R*^2^ = .109, Δ*R*^2^ = .00, Δ*F*(1, 1772) = 0.44, *p* = .51). This result contradicts Hypothesis 4b. Therefore, the predictors of the third step were not included for the interpretation of the regression coefficients.

First, gender (β = .07, *p* = .006), cycling fandom (β = .25, *p* < .001), age (β = –.05, *p* = .036) and education (β = –.06, *p* = .015) were significant predictors. This means that men include international cyclists more in their consideration of a *flandrien* than women, and older and higher-educated Flemings include international cyclists less in their consideration of a *flandrien* than younger and lower educated Flemings, respectively. Furthermore, the more strongly respondents claim to be cycling fans, the more they include international cyclists in their consideration of a *flandrien*.

As expected, ethno-cultural identity representation emerged as a significant, negative predictor (β = –.15, *p* < .001). That is, the more Flemings endorse an ethno-cultural identity representation, the less they will include international cyclists in their consideration of a *flandrien*. This finding is in line with Hypothesis 2b. In line with Hypothesis 3b, Flemish identification was no significant predictor in this model (β = –.03, *p* = .23).

### Inclusion of international cyclists as flandriens: cyclists list

Step 1 revealed that the background variables accounted for 14.8% of variance in the average score given to the four international cyclists (*R*^2^ = .148, *F*(4, 1720) = 74.50, *p* < .001). The results of step 2 revealed that, when the background variables were statistically controlled for, the addition of ethno-cultural identity representation and Flemish identification contributed significantly to the inclusion of international cyclists as *flandriens* (*R*^2^ = .151, Δ*R*^2^ = .003, Δ*F*(2, 1718) = 3.19, *p* = .041). The results of step 3 showed that, when controlling for the background variables and ethno-cultural identity representation, the interaction between ethno-cultural identity representation and Flemish identification did not contribute significantly to the inclusion of international cyclists as *flandriens* (*R*^2^ = .151, Δ*R*^2^ = .002, Δ*F*(2, 1624) = 2.39, *p* = .092). This result contradicts Hypothesis 4b. Therefore, we further only discuss the regression coefficients for the predictors after the second step.

First, gender (β = .07, *p* = .002), cycling fandom (β = .35, *p* < .001), age (β = .12, *p* < .01) and education (β = –.06, *p* = .008) emerged as significant predictors. This means that men and older respondents include international cyclists more in their consideration of a *flandrien* than women and younger respondents, respectively, and that higher educated respondents include international cyclists less in their consideration of a *flandrien* than lower educated respondents. Furthermore, the more strongly respondents are cycling fans, the more they include international cyclists as *flandriens*.

As expected, ethno-cultural identity representation was a significant, albeit modest, negative predictor (β = –.06, *p* = .016). That is, the more Flemings endorse an ethno-cultural identity representation, the less they include international cyclists in their consideration of a *flandrien*. This finding is in line with Hypothesis 2b. Again, Flemish identification was no significant predictor in this model (β = .01, *p* = .66), which confirmed Hypothesis 3b.

## Discussion

The present study aimed to offer further insights into the current meaning of the term *flandrien* for Flemish citizens and its relation with the way in which Flemish identity is represented. For this reason, we decided to use a quantitative approach, thereby extending previous qualitative research on the term *flandrien*. More specifically, this research investigated the relation between the identity representation of Flemings and their interpretation of the historical cycling term *‘flandrien’* as an important symbol for the Flemish identity.

In line with a qualitative study from Knuts et al. (2011), we revealed that the majority of the Flemings disagree with the contemporary depiction of a *flandrien* by the media as being regionally unbounded. It should be noted that the proportion of Flemings who disagree with the statement that a *flandrien* can also be foreign cyclist was lower in the present study compared with the proportion found by Knuts et al. (2011). Nevertheless, using a much larger sample, we did replicate their finding that most Flemings still have a regionally restrictive definition of this term and consider a *flandrien* as a non-international cyclist, thereby confirming Hypothesis 1.

An important finding was that the more Flemings endorse an ethno-cultural identity representation, the more they protect the meaning of a *flandrien* in terms of regional exclusivity against change. In line with Hypotheses 2a and 2b, we observed that the more strongly Flemings endorse an ethno-cultural representation of their Flemish identity, the more they consider a *flandrien* as an exclusively Flemish cyclist and the less they include international cyclists in their consideration of a *flandrien*. This finding clearly demonstrates that Flemings who endorse an ethno-cultural identity representation are opposed to change the meaning of the term *flandrien*. In the context of the SIA this study demonstrates that sports can create in-group prototypes. More specifically, a *flandrien* is considered as an in-group prototype with which Flemings appear to agree. This finding underlines the important role of sports in the process of creating and enhancing identities. Moreover, this study shows how identity representations can influence on (intergroup) attitudes and norms in sports contexts.

Flemish identification positively predicted the consideration of a *flandrien* as a Flemish cyclist, thereby confirming Hypothesis 3a. Considering that a *flandrien* is generally regarded as a positively valued term, Flemings who strongly identify themselves as Flemish want to ascribe the term to their ingroup, thereby aiming to verify the positive view they (want to) have on their group membership. This finding is in line with previous findings regarding the positive relation between ingroup identification and ingroup favouritism (e.g., Castano et al., 2002).

Flemish identification was not related to the inclusion of international cyclists as *flandriens*. This was in line with several studies indicating that ingroup identification is not necessarily positively related to explicit outgroup derogation (e.g., Pehrson, Brown & Zagefka, 2009; Pehrson, Vignoles & Brown, 2009). In sum, the abovementioned finding underline the strength and importance of a social identity perspective on group processes, as apparently the same mechanisms are at work in very divergent settings, ranging from politics concerning immigrants to sport settings.

It should be noted that in contrast with Hypothesis 4, Flemish identification did not moderate the relation between an ethno-cultural identity representation and the attribution of a *flandrien* in terms of regional exclusivity. This lack of interaction between identification and ethno-cultural representation contrasts the findings from Pehrson, Vignoles, and Brown (2009). One possible explanation for this discrepancy is that individuals who endorse an ethno-cultural identity representation might have a conservative attitude in general. Consequently, they might consider a *flandrien* as a cultural symbol that they do not want to see changed because they are against change in general, regardless whether it is important for the Flemish identity or not. Therefore, an interesting avenue for future research is to disentangle the effects of an ethno-cultural identity representation and a conservative attitude in general.

Two additional findings are worth discussing. A first interesting finding is that cycling fans are less regionally exclusive in the consideration of a *flandrien* as compared to non-cycling fans. Cycling fandom was the strongest predictor amongst all background variables. A possible explanation for this relation is that cycling fans spend more time following cycling in sports media, which depict a *flandrien* as not exclusively Flemish. In turn, this larger exposure to the perception of a *flandrien* by the media could have an influence on their own perception. A second possible explanation is that because of their larger interest in sports, cycling fans consider a *flandrien* as a term that is strictly related to sports. This would isolate the term from any association with regional exclusivity or identity. In this respect, it should also be noted that sport contexts can trigger both the separating as well as the uniting function of identities. In fact, according to SIA (Haslam, 2004), the specific level at which individuals define themselves at a particular moment – and thus act accordingly – depends on the salience of their social identities. This salience is in turn determined by the perceived fit of a social identity with the context and by the readiness of the perceiver to identify with this identity. For example, because many Flemish flags are displayed along the route of each Tour of Flanders, this environmental cue will enhance the likelihood that individuals will define themselves as a Flemish person during that race because this social identity then fits with the context. In addition, this fit is more likely to be perceived by individuals who already value this social identity (i.e. high identifiers). These high identifiers are more ready to apply this self-categorization because it is more available in their minds.

In other words, self-categorization is a dynamic and complex process. The level at which a social identity is triggered depends both upon the specific context and upon the individual’s predisposition. In the case of the cycling fans in our questionnaire, they were probably more likely to define themselves (and the cyclists) as cycling fans than as Flemish persons, because they were addressed as cycling fans to fill in the questionnaire. As a consequence, their social identity of ‘cycling fans’ was more salient to them than their social identity of a Flemish person.

A second interesting finding is that men are less regionally exclusive in the use of the term *flandrien* than women. However, additional analyses showed that on average, men (*M* = 4.47) were stronger cycling fans than women (*M* = 3.73) (*t*(2006) = –13.44, *p* < .001). For this reason, the aforementioned explanation with respect to the relation between cycling fandom and regional exclusivity can also account for the finding that men are less regionally exclusive than women.

### Strengths and limitations

One of the major strengths of the present study is that we adopted a quantitative approach to investigate the importance of a *flandrien* in symbolizing a Flemish identity. By using regression analyses on a large-scale internet-survey data, the present study extends previous studies, which only used qualitative material (e.g., interviews, primary sources or secondary literature) to study this topic. This approach, including the large sample size, elaborates previous qualitative findings that simply describe the existence of differences in perception of a *flandrien* (Knuts et al., 2011), by including a theoretical framework in the shape of SIA to explain these observed differences. Another strength is that the results revealed similar predictors for both operationalizations of the inclusion of international cyclists in the consideration of a *flandrien*. The one-item scale (‘a *flandrien* can also be a foreign cyclist’) can be considered to be more abstract than the concrete list of cyclists. The fact that we found similar relations between these outcomes on the one hand and Flemish identification and ethno-cultural identity representation on the other hand testifies to the validity of our findings.

A first limitation of this study addresses the relatively small effect sizes (0.3%–6%) of our focal predictors: ethno-cultural identity presentation and Flemish identification. This means that although endorsing an ethno-cultural identity representation and Flemish identification did predict the interpretation of a *flandrien* in terms of regional exclusivity, this effect was not particularly pronounced. In line with the findings regarding the other predictors, this indicates that other factors, such as cycling fandom, have a stronger influence on the perceived regional exclusivity of a *flandrien*.

A second limitation regards one of this study’s operationalizations of the inclusion of international cyclists as *flandrien*. That is, respondents had to rate to which they considered four international cyclists as a *flandrien*. We chose only these four cyclists because we expected them to be largely known amongst Flemings and demonstrate the typical ‘Briek Schotte-characteristics’. This enables to really capture the regional aspect of these cyclists and limits the possible confounding influence of these characteristics although we acknowledge that this choice is subjective, but still based on the opinion of cycling experts.

A third limitation of this study is that an ethno-cultural identity representation, as a combination of an ethnic and cultural identity representation, has never been studied as an identity representation in itself. Traditionally, the most occurring distinction is between a civic and ethnic identity representations. More recently, Kymlicka (2001) argued for the conceptualization of a cultural identity representation. A study of Reijerse et al. (2012) also found evidence for the distinction between an ethnic, civic and cultural identity representation in the context of anti-immigrant behaviours. However, these authors reported a positive correlation between their ethnic and cultural identity representation scales. In addition, the items of Reijerse et al. (2012) that are used in this study are supposed to measure an ethnic and cultural identity representation. However, the results of the principal component analysis in our study showed that these items loaded highly on one factor and therefore could not be distinguished from each other. Together, these findings are not surprising because of the aforementioned overlap in terms of conceptualization between an ethnic and cultural identity representation. Therefore, it seems legitimate to conceptualize an ethno-cultural identity representation as an identity representation that is conservative and opposed to change in identity-relevant elements.

## Conclusion

To conclude, this study demonstrates that the expansion of the term *flandrien* in terms of regional exclusivity is not shared by all Flemings and is influenced by their identity representation. On the one hand, the results of this study reveal that a *flandrien*, a historically contested cycling term, is still considered as an important symbol for the Flemish identity. On the other hand, adhering to an ethno-cultural identity representation seems to influence Flemings’ perception of this identity-relevant symbol in terms of changeability. In spite of his heroic performances and match with behavioural characteristics, Flemings with an ethno-cultural identity representation will less likely consider the cycling champion Cancellara as a *flandrien*, simply because he is not a Fleming.

## Additional File

The additional file for this article can be found as follows:

- pb-58-1-358-s1.sav
  **Supplementary file 1** Dataset. DOI: https://doi.org/10.5334/pb.358.s1
